# DC SIGNR silencing reduces HIV-1 infection in Dendritic cells

**DOI:** 10.1186/1471-2334-14-S3-O8

**Published:** 2014-05-27

**Authors:** Omkar Chaudhary, Sanjeev Kumar, Heena Aggarwal, Muzamil A Makhdoomi, Jasbir Singh, Anjali Hazarika, Kalpana Luthra

**Affiliations:** 1 Dept. of Biochemistry, All India Institute of Medical Sciences, New Delhi, India; 2 Dept. of Biochemistry, Kurukshetra University, Kurukshetra, India; 3 Blood Transfusion Services, Cardiothoracic and Neurosciences Center, All India Institute of Medical Sciences, New Delhi, India

## Background

Dendritic cells (DCs) capture HIV-1 from periphery via DC SIGN/R receptors and transfer to T cells. It has been reported that siRNA directed against DC SIGN significantly inhibits HIV infection of DCs. In this study, the expression of DC SIGN/R and its role in HIV-1 infectivity of DCs were assessed.

## Methods

DCs were cultured from monocytes from healthy donors and infected with HIV-1 Indian clade C virus. DC SIGN/R expression on DCs was determined by RealTime PCR. The effect of down regulation of this receptor in DCs using DC SIGN/R siRNA on the infection with HIV-1 was assessed. Statistical significance was calculated by Student’s t test.

## Results

High expression of DC SIGN/R was observed on DCs infected with HIV-1 (*p*= 0.01). DC sIGNR expression in DCs was maximally downregulated by siRNA at 24 hrs (*p*=0.002) and was associated with significant reduction in expression of CD40 (*p*= 0.003), CD80 (*p*=0.008), CD86 (*p*=0.007) and P38 MAPK (*p*=0.005). Transfection of DC SIGNR on DCs at 24 hours followed by infection with clade C HIV-1 demonstrated lower levels of p24 compared to that in untransfected DCs (*p*=0.0008).

## Conclusion

Data demonstrates that down regulation of DC SIGNR expression on DCs decreases activation that in turn may inhibit DC T cell interactions needed for progression of HIV-1 infection. Reduced p24 antigen production may be mediated by P38 MAPK inhibition. Our finding suggests that blocking DC SIGNR expression on DCs may prevent initial binding of HIV-1 and serve as a first step in prevention of HIV-1 infection.

